# Embolization of a Fractured Peripherally Inserted Central Catheter to Pulmonary Arteries: A Sporadic Life-Threatening Phenomenon

**DOI:** 10.7759/cureus.43044

**Published:** 2023-08-06

**Authors:** Amitabh Kumar Upadhyay, Binayendu Prakash, Shashank Shekhar, Abhishek Kumar, Aaditya Prakash

**Affiliations:** 1 Medical Oncology, Tata Main Hospital, Jamshedpur, IND; 2 Cardiology, Tata Main Hospital, Jamshedpur, IND; 3 Medical Oncology, Meherbai Tata Memorial Hospital, Jamshedpur, IND; 4 Nuclear Medicine, Tata Main Hospital, Jamshedpur, IND; 5 Radiation Oncology, Tata Main Hospital, Jamshedpur, IND

**Keywords:** cancer, fracture, cvc, embolization, picc

## Abstract

The peripherally inserted central catheter (PICC) is a non-tunneled central venous catheter placed in the upper limb venous system, mainly in the basilic vein, and the tip terminates in the superior vena cava (SVC). A PICC is a preferred modality of central venous access in oncology, as it is associated with minimal discomfort and can be kept in situ for up to one year. Despite multiple advantages, it is also associated with complications. Fracture and migration are rare but potentially serious complications that can lead to arrhythmias, cardiac perforation, cardiac tamponade, pulmonary embolism, and sepsis. The migrated PICC fragment can be retrieved using percutaneous techniques, which have a high success rate of excess, with minimum complications. In our patient of adenocarcinoma gastroesophageal junction, the fractured and migrated PICC to pulmonary arteries was retrieved using the balloon catheter method. With more and more cancer patients using PICCs for chemotherapy administration, healthcare workers must be aware of the standard and sporadic complications of PICCs. Care of the PICC is crucial, and any lapse may lead to fracture and embolization, which is a potentially life-threatening complication. This case highlights the importance of healthcare persons being aware of the possibility of catheter embolization and methods to prevent and mitigate this phenomenon.

## Introduction

The peripherally inserted central catheter (PICC) is a non-tunneled central venous catheter (CVC) placed in the upper limb venous system, mainly in the basilic, median cubital, or cephalic veins. PICCs are 50- to 60-cm-long single, double, or triple lumen catheters that traverse through the brachial, axillary, and subclavian veins, and the tip terminates at the superior vena cava (SVC) or cavo-atrial junction [1]. The basilic vein is usually preferred due to its larger diameter, superficial location, the fewest valves, straighter route, and requiring a shallower angle of needle insertion while inserting a PICC, compared to other veins [1]. It is usually inserted by trained physicians or nurses using the blind technique or under ultrasound guidance and fluoroscopy supervision.

Peripheral intravenous access is a temporary venous access complicated by superficial thrombophlebitis, risk of extravasation, discoloration of skin, and cellulitis, and hence not suitable for long-term treatment in oncology. PICC is a preferred modality of central venous access in cancer cases, as they require long-term venous access. It is associated with minimal discomfort and can be kept in situ for up to one year, decreasing the pain and anxiety associated with repeated venipunctures [1-3]. PICC is suitable for cases with difficult peripheral venous access, the requirement of long-term intravenous (IV) medications like antibiotics, and antifungals, requiring administration of vesicant or irritant chemotherapies, and requiring an infusion of total parenteral nutrition. It is also suitable for blood component infusions, frequent blood sampling, patients with coagulopathies, and those with anatomic abnormalities in the neck or thorax that make CVC insertion difficult [1-3]. Placement in the antecubital fossa or at mid-arm carries the critical advantage of moving the exit site of the catheter away from endotracheal, oral, and nasal secretions.

The life of a PICC in situ depends upon the material, catheter stabilization, patient compliance, and nursing competence in handling and maintenance [3-8]. Transparent bio-occlusive dressings are applied over PICCs to prevent infection [3-8]. A PICC requires regular insertion site dressing and weekly catheter flushing [3-8]. The other long-term CVCs used in oncology practices are Hickman (cuffed) catheters, subcutaneously implanted chemo ports, and centrally inserted central catheters (CICCs), or central lines. Central lines are unsuitable for long-term venous access for oncology. In the oncology settings, PICCs are preferred as they can be safely used even in patients with thrombocytopenia where other central venous catheters are difficult to insert [3-8]. PICCs are associated with a low rate of complications, like occlusion (8.9%), accidental removal (8.9%), catheter-related infections (6.3%), venous thrombosis (1.6%), and hematoma (1%) [6]. Catheter fracture and embolization is a sporadic phenomenon noted in less than 1% of such cases, and is associated with significant morbidity and mortality and necessitates the need for prompt removal. Here, we report a case of PICC fracture, which had embolized to pulmonary arteries and was successfully removed by our interventional cardiology team.

## Case presentation

**Figure 1:**
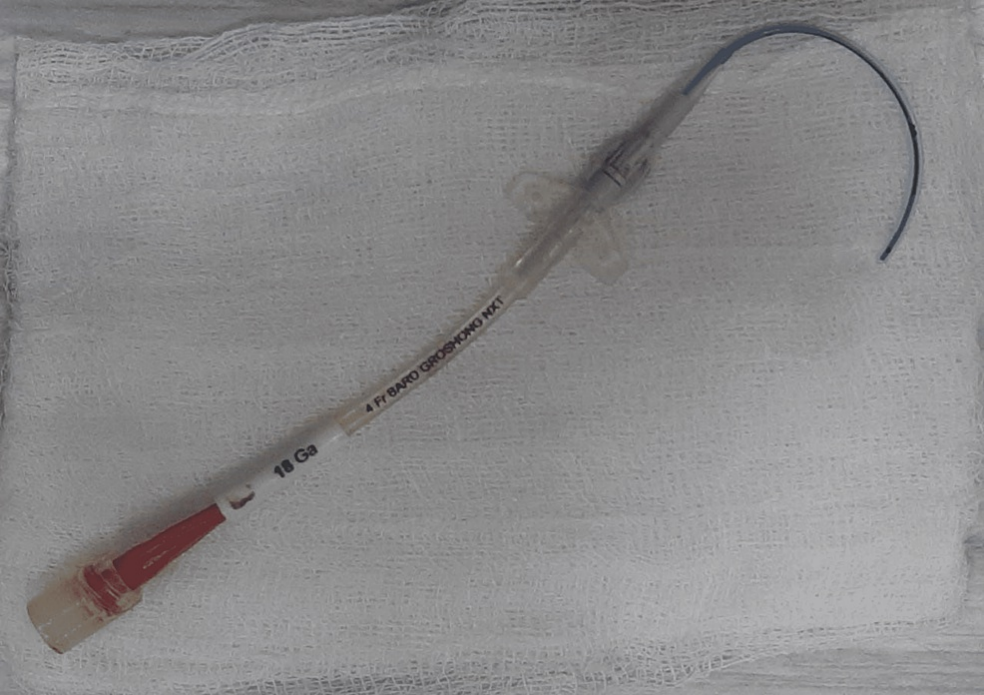
The broken peripheral part of the PICC PICC, peripherally inserted central catheter

A 76-year-old male patient presented to our outpatient department (OPD) with complaints of abdominal pain, loss of appetite, and melena, in October 2022. Upper gastrointestinal endoscopy (UGIE) showed ulcero-infiltrative Siewert type III growth at the gastroesophageal (GE) junction extending to the cardia and part of the adjacent fundus. A biopsy confirmed moderately differentiated adenocarcinoma, negative for HER2/neu and microsatellite instability (MSI) on immunohistochemistry. Positron emission tomography/CT (PET-CT) showed hypermetabolic circumferential mural thickening involving the GE junction, cardia, and adjacent fundus of the stomach with hypermetabolic lymph nodes in lower peri esophageal, retrocrural, aortocaval, retroaortic, and paraaortic regions. He had normal blood counts, kidney and liver functions, and Eastern Cooperative Oncology Group (ECOG) performance status 1. A 4 French single-lumen silicone PICC was inserted in the right basilic vein under ultrasound guidance (in December 2022) with the tip at the cavo-atrial junction, confirmed by post-procedure chest X-ray. There was no evidence of any knots or kinks at the tip. He was started on palliative chemotherapy with the FLOT regime (5-fluorouracil, leucovorin, oxaliplatin, docetaxel). The patient accidentally removed the PICC, and a new 4 French single-lumen silicone PICC was inserted in the right basilic vein under ultrasound guidance in February 2023 with the tip at the cavo-atrial junction, confirmed by the post-procedure chest X-ray. The PICC was used for chemotherapy without any problems, and regular weekly dressing and flushing were done. The patient came to the OPD in June 2023 with a complaint of a broken PICC and showed us the 12-cm-long peripheral broken part of the PICC (Figure 1). In the patient’s language, the fractured piece of the PICC came out automatically while he was traveling. He was otherwise asymptomatic.

**Figure 2:**
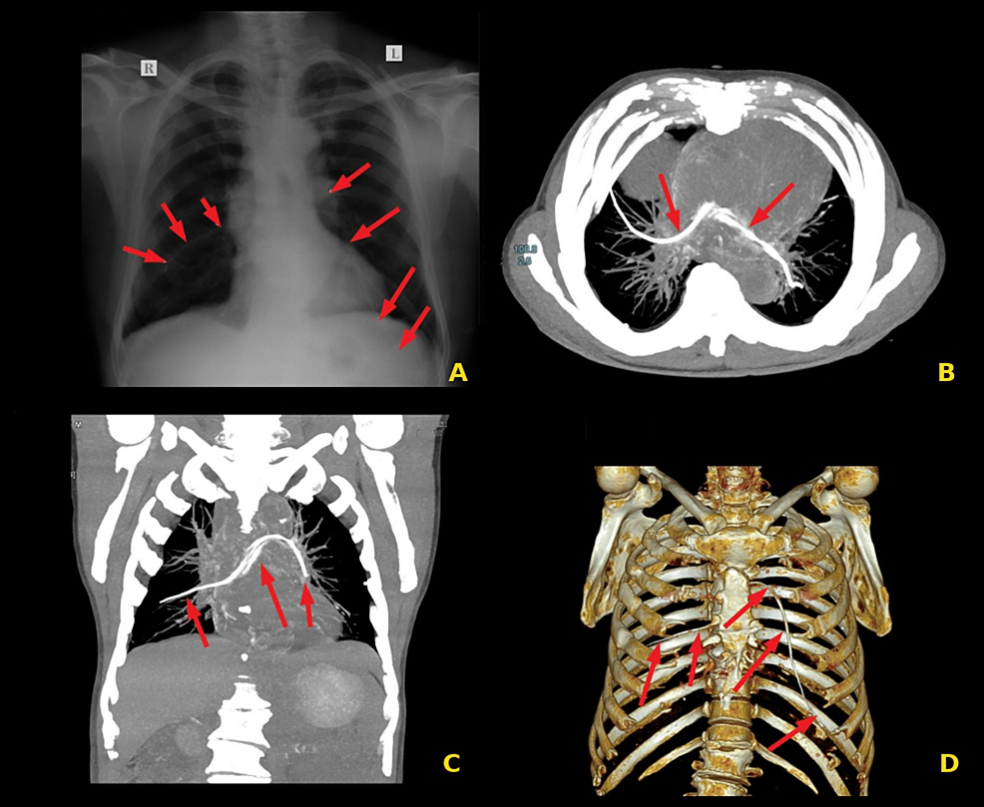
(A) Chest X-ray showing broken PICC line fragments (arrows) in bilateral lungs extending into peripheral pulmonary vasculature; (B) axial CT image revealing the migrating PICC in the right pulmonary artery's right middle lobe segmental branch (arrows). It is also seen extending into the left lower lobe segmental branch of the left pulmonary artery (arrows). The proximal end of the broken PICC was noted in the region of the main pulmonary artery bifurcation; (C) coronal CT image revealing the migrating PICC in the right middle lobe segmental branch of the right pulmonary artery (arrows). It is also seen extending into the left pulmonary artery (arrows); (D) 3D reconstructed CT images revealing the migrated PICC extending into the segmental branches of bilateral pulmonary arteries (arrows) PICC, peripherally inserted central catheter

A chest X-ray was done, which suggested linear density in the bilateral lower pulmonary parenchyma (Figure 2A). Contrast-enhanced CT (CECT) of the thorax was performed, which suggested an embolism of the PICC into the right middle lobe segmental branch extending into the left lower lobe segment of the left lung (Figures 2B-2D). His blood reports showed a hemoglobin value of 10.1 g/dl, platelet count of 1.65 lacs, and normal kidney and liver functions. Urgent cardiology intervention was requested for retrieval of the embolized fractured segment of the PICC.

**Figure 3:**
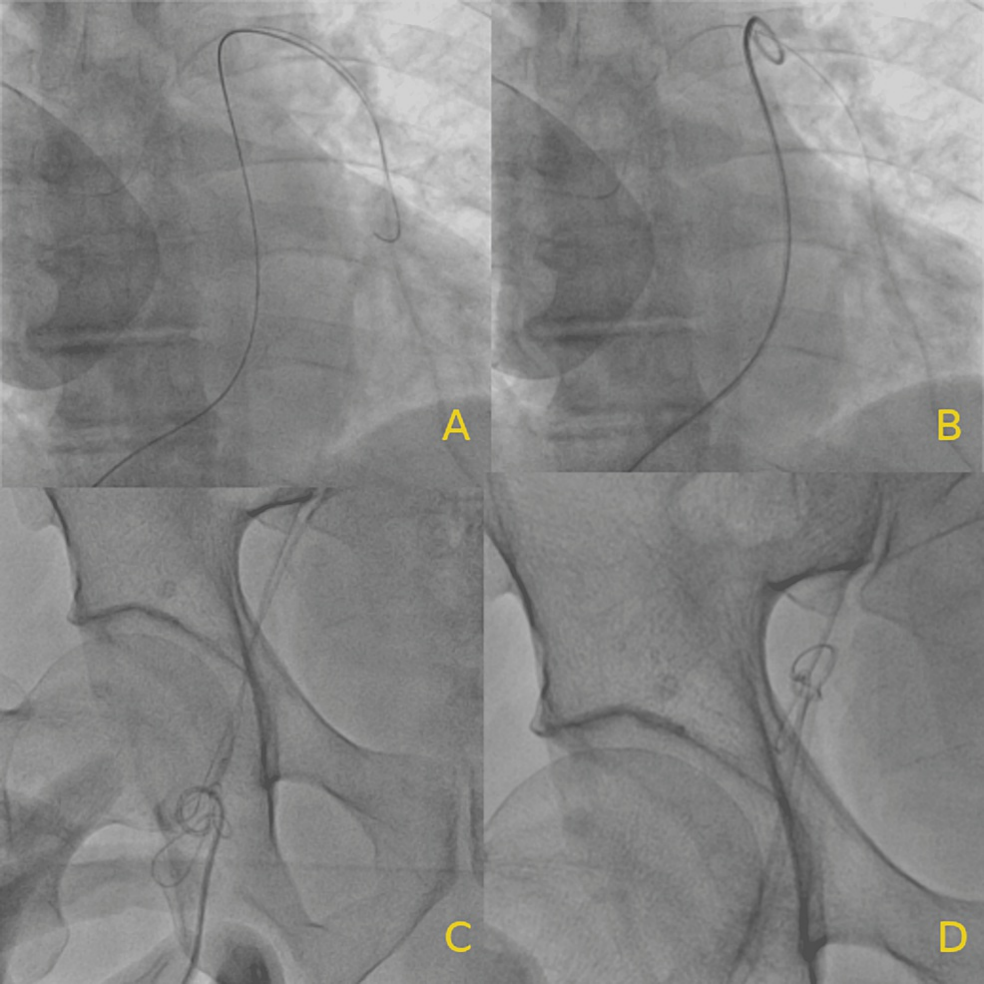
(A) Guidewire positioned in the left pulmonary artery; (B) pigtail catheter hooking the PICC; (C) PICC entangled with the pigtail catheter being pulled out; (D) loop snare catching the entangled PICC PICC, peripherally inserted central catheter

**Figure 4:**
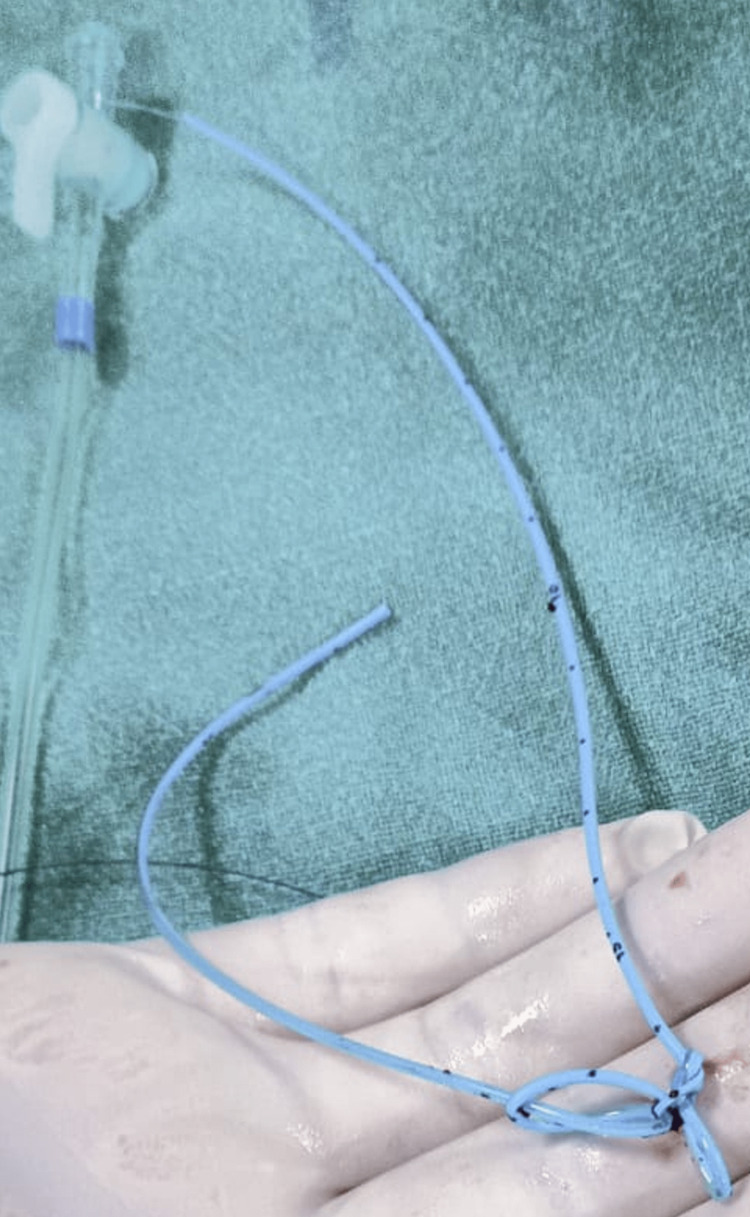
Removed PICC PICC, peripherally inserted central catheter

The PICC was seen under a fluoroscope to be migrated to the right segmental level pulmonary artery with the distal end in the left pulmonary artery. A French sheath was inserted in the right femoral vein, and a guidewire was maneuvered into the left pulmonary artery. Over this, a 6 French pigtail catheter was traversed to the left pulmonary artery. The pigtail was rotated to hook the fragmented distal end of the PICC, and once it was grasped, the pigtail was rotated clockwise several times to entangle it over the pigtail. It was pulled to the inferior vena cava (IVC), and the whole system, including the femoral sheath, was pulled out, but while drawing, the distal end of the pigtail catheter became straight, and the PICC retained in the IVC. A second puncture was done in the right femoral vein, and again the sheath was inserted. This time a 6 French loop snare was used to catch the PICC fragment, and now the whole system was pulled out, including the fractured part of the PICC (Figures 3A-3D, Figure 4).

There was no evidence of any arrhythmia during and after the procedure. The patient was asymptomatic after the procedure and was shifted to the oncology department for further management.

## Discussion

PICCs are very suitable for oncology patients but are associated with complications such as catheter-related infections, leakage, kinking, phlebitis, thrombosis, accidental removal, fracture, and migration [2-8]. The long-term placement of PICCs may also be related to an increased risk of complications like pneumothorax, arterial puncture, haemothorax, arrhythmias, and nerve injuries [2-8]. Fracture and migration are rare but potentially serious complications that can lead to arrhythmias, cardiac perforation, cardiac tamponade, pulmonary embolism, and sepsis. Most patients are asymptomatic, but potentially lethal sequelae can develop [4-8]. The PICC fragments migrate along the bloodstream and can lodge in the superior vena cava (15.4%), the right atrium (27.6%), the right ventricle (22%), and pulmonary arteries (35%) [7]. Sometimes, the migrated PICC adheres to the endocardium, leading to uncontrolled arrhythmias. It can rarely cross to left cardiac chambers through the unclosed foramen ovale, leading to systemic embolism and further complications. Factors like PICC's weight, length, and material stiffness influence its final site of lodgement [2-8].

Linz et al. published the first report describing the embolization of a fractured PICC in 1994 in Japan [9]. The accurate incidence rate of PICC fractures still needs to be discovered due to a lack of long-term data and large prospective studies. Chow et al. hypothesized the role of PICC fatigue responsible for the fracture, and the average time of PICC fracture while in situ was 93 days [10]. Similar cases were reported by Joga et al., Krishnan et al., Chandrashekhara et al., Sood and Srinivasan, and Pande et al. from India [11-15]. The embolization of a fractured PICC was reported in children by Fang et al. and Hu et al. [16,17]. Catheter fatigue was also hypothesized as the probable reason for fracture in our case. The patients are given instructions regarding PICC care at home, but that is not followed religiously in rural populations, and some patients continue to do their physical work despite having a PICC in situ. The same was seen in our case as the patient continued his routine activities. The duration of the catheter in situ, quality of care, patient awareness, and material of the PICC are other factors associated with the fracture and migration. Catheter fracture can happen during insertion if excessive syringe pressure is applied, during removal, or because of tractional force on the catheter hub junction. When attempting to remove a PICC, if there is resistance, repeated hot soaks can be applied to the insertion site to expand the veins. No attempt should be made to pull the catheter forcibly. After the removal of PICC, its intactness should be meticulously checked by healthcare workers. The in situ time of a PICC is a critical factor for the fatigue and fracture of the PICC. However, routine removal or reinsertion is not recommended solely because of duration, as it is associated with risks and avoidable costs to patients.

A PICC is made of silicone or second- or third-generation polyurethane. Polyurethane PICCs have thinner lumen walls and larger internal diameters, which leads to higher flow rates; they have a lesser probability of fracture of the catheter but are associated with a higher incidence of thrombosis compared to silicone PICCs. Silicone is more biocompatible than most polyurethane catheters and so more suitable for long-term use with a comparatively higher risk of catheter fracture. In a review by Seckold et al., lower infection rates, dislodgement, thrombus, and fracture rates were found with polyurethane PICCs [18]. Mou et al. have shown a higher incidence of catheter breakage with silicone catheters [19]. In a study by Ong et al., an increased incidence of phlebitis but equal catheter fracture and migration rates were reported in the silicone catheters compared to the polyurethane catheters [20].

The migrated PICC fragment can be retrieved by percutaneous techniques, which have a more than 90% success rate with minimal reported complications. Interventional techniques like loop snares, hooked guide wires, forceps, basket catheters, and Fogarty balloon catheters are used to retrieve the dislodged catheter [21]. The loop snare method is mainly used as it is safer and more effective. While using a loop snare to retrieve the migrated PICC, the loop snare must grasp the tips of a catheter. If the tip of the catheter is lodged at the wall of the blood vessels, or a complex plane, it becomes very challenging to grasp it successfully. It is difficult to control the movement of the loop in the pulmonary artery and its branches, so it becomes challenging to grasp the catheter tip if it migrates to that level. The migrated PICC was at a similar location in our case, so it was difficult to apply the loop snare method for the same. Hence, the pigtail catheter method was used to retrieve the migrated PICC. A novel two-step method was described in five cases by Peng et al., in which the fractured PICC segments located in the cardiac chambers or the pulmonary artery were removed using a pigtail catheter combined with a vena cava filter retrieval set [22]. In this method, the migrated catheter is grasped using a pigtail catheter. It is brought to the IVC, where the catheter is held and removed using a filter retrieval set. However, the retrieved catheter may be lost during the transition between the two steps, which increases the overall procedure duration. An endovascular technique with a loop snare and a suture was reported by Teragawa et al. for removing a fractured PICC in the pulmonary artery [23]. Although this technique is a valuable method for controlling catheter movement, it may be associated with a potential for vessel wall injury. Yen et al. reported the goose-neck snare method for removing a migrated catheter with its free end in the pulmonary trunk in 13 patients with CVC fragment embolization, including four PICCs [24]. If the migrated catheter is stuck in the trabecula of the right ventricle, a floppy guide wire can be used to grasp it, as the loop snare cannot hold it. The guidewire can be inserted through the other vein to cross the catheter and hold its tip by the loop snare. Another two-step method was successfully used by Sood and Srinivasan for removing an embolized PICC from the pulmonary artery. A catheter was used to hook and pull the embolized PICC into the lower segment of the IVC, where it was grasped by a loop snare and removed successfully [14]. Pande et al. applied a novel method with the help of biopsy forceps for retrieving the migrated PICC from the right atrium. The PICC loop was hooked by a catheter and dragged to the inferior vena cava, where the biopsy forceps grabbed it and was successfully removed [15]. However, in rare cases, surgical intervention may be required. Usually, a patient may suffer from transient arrhythmia while undergoing the procedure that disappears soon after catheter removal.

Regular maintenance with weekly flushing and dressing of the PICC is crucial, which is to be done by specially trained nurses under a strict aseptic technique. Patients and their family members should be educated regarding the care and protection of lines, especially during discharge. Patients should not use the limb with the PICC for heavy weightlifting, strenuous exercise, hectic games, or some other heavy work. While taking a bath, a fresh film can be used to wrap the limb with the catheter to protect it. Patients should also be taught the possible complications, including PICC breakage with symptoms and signs, and to report immediately to the healthcare providers in the case of breakage.

## Conclusions

PICC is a preferred CVC in oncology, but it has its known common and rare complications, which should be known to healthcare workers. Care of the PICC is a very crucial aspect for healthcare workers and patients. Any lapse in care may lead to fracture and embolization, which is a potentially life-threatening complication. Loop snare is the most common method of retrieval of migrated CVCs but is challenging to use in pulmonary arteries. Of late, most of the oncology research has been on novel targeted therapies, immunotherapies, surgical techniques, etc., leading to lesser attention from healthcare workers on CVC care and its possible complications. This case reiterates that oncologists and healthcare workers should consider having specially focused sessions with their team and patients about the basics of CVC care and its potential complications. This report provides a detailed literature review of similar reported cases and a detailed discussion of the available and performed retrieval methods. This case emphasizes the importance of having interventional cardiology and radiology facilities near high-volume oncology centers so that immediate intervention can be done without wasting valuable time traveling to other cities.
